# The Value of Electricity in Placenta Prævia

**Published:** 1887-02

**Authors:** W. M. Powell

**Affiliations:** Albany, Texas


### THE VALUE OF ELECTRICITY IN PLACENTA PRÆVIA.

*By W. M. Powell, M. D. Albany, Texas.*

(For Daniel's Texas Medical Journal.)

ALREADY some little has been written in regard to the effects of this agent in post partem hemorrhage, and I see no good reason why it should not be a potent method in arresting anti-partem hemorhage.

It is clear to my mind, that anything that will produce, almost, instantaneous contractions of the uterus in cases as formidable as the one under consideration, is the thing to be employed if possible.

My experience with electricity in general obstetric practice has been such as to justify its use in almost every case, much more when we are called upon to deal with cases so fraught with danger as the one I shall now refer to.

Mrs. S., aet.—very fair complection, the mother of seven living children, (two miscarriages,) complained of having head ache with slight fever, very nervous, etc., on the 6th of December last. That night she had a very slight discharge of blood per vagina, just sufficient to stain her linen. Her husband met me on the street the following forenoon 'and told me her condition, with the request that I should call some time in the afternoon to see her. They did not wait, however for me to call at my leisure but sent for me very hurriedly some two hours after the interview. I found Mrs. S. sitting up, but she informed me that she was loosing considerable blood, and that her back was hurting very badly. I immediately had her take her bed and at once made a digital examination, removing several small clots as I did so, but could not determine the true nature of the case as there was very little dilatation. I enjoined perfect rest in the recumbent position and prescribed secale and viburnum, and instructed her husband to send for me promptly if she should get worse. I had time to call again at 6 p. m. without being sent for, and found her seemingly quite comfortable. At 9 o'clock I was called to go in great haste, the messenger stating that "she was nearly dead." The distance being short I soon arrived, and fcund her quite exhausted from the most frightful hemorrhage I have ever seen in eleven years of active practice. I very promptly cleared the vaginia again of a great mass of clots and found sufficient dilatation to confirm me in my opinion from the start, that was, that of a placental presentation, for I could easily detect a portion of the afterbirth a little in advance of what I supposed to be the head, for, to be sure I could not be certain. However I lost no time in tamponing, with the view of course, of suppressing the flow (which appeared to be a continuous stream) until an engagement of somekind should take place, but this did no good that I could discover at all. I then sent a runner for my battery, and an other for my friend Dr. M., for I saw that my patient was rapidly sinking. The battery being much nearer soon came, and as quickly as I could possibly set it in running order I applied the positive electrode to the sacro-lumbar region, and the negative all over the abdominal points,and in less than two minutes the hemorrhage ceased, and the constant hurting in her back was relieved. Some fifteen minutes later Dr. M. came, made an examination, but did not detect the placenta as I had done, and as all hemorrhage had stopped he took his leave, stating that she might possibly, go on to full time, (this being as nearly as could be determined eight months.)

Mrs. S. fell asleep for a short time, but in just one hour from the time I checked the flow, it recurred with, seemingly double force and quantity. I again applied the electric current as before, and in equally as short a time controlled it. I then made another examination and could easily make out the presentation (vertex) and quite a surface of the placenta just in advance. Having my forceps at hand, I naturally concluded to apply them and deliver at the earliest practicable moment, as my patient was as near pulseless, and at death's door as I ever saw any one to recover. I again sent for

Dr.M. as I wanted him to be sure that it was indeed a case of placenta prævia, and help me save her life, if possible. He soon was on hand and after a second careful examination confirmed my dignosis, and fully agreed with me in my idea of instrumental delivery as soon as there was sufficient dilatation and advancement of the head to warrant it. By this time the head was pretty well engaged and actual labor set in rather vigorously, and the amniotic waters allowed to escape by rupturing the sac.

No more hemorrhage of any special consequence occurred,and at half past three a. m. I delivered her of a very well developed, dead male child. I will state in this connection, that, according to her statement, no fœtal movement was felt after 10 o'clock in the evening, and all efforts to resuscitate the child of course proved futile. In a reasonable length of time the afterbirth was expelled, presenting a torn, bleeding surface, or edge, some five inches in length, which of course accounted for such a fearful hemorrhage.

The position of the presenting placenta was to the right, the lower end of the rent diping down as it were towards the hollow of the sacrum, the upper extending above the sacro-illiac sympyhsis, overlaping something like one-half the orifice; hence it will be seen that so much of the laceration extended below the bony structure that it afforded a free source for continuous hemorrhage, while that part above exposed numerous vessels to augment the flow at each contraction and dilatation; consequently the head, naturally had to come well down, pressing the soft parts before it could serve as a complete plug.

Dr. M. at his first visit insisted on giving opiates which I declined to agree to, as past experience, with her, had shown me that she could not bear opium in any form; and besides, opium, like ergot, has its particular place in treating diseases, and since ergot had failed to control hemorrhage in this case, I could not hope for opium to do so.

The question might very properly be asked, how could electricity have controlled this violent hemorrhage in so short a time after the other means employed so uterly failed ? I have but one theory to advance. We know that ergot does not at all times act uniformly on the transverse and longitudinal fibers of the uterus, and upon this hypothesis electricity should claim the preference since Beard, Rockwell, Bartholow, and Althus, Morgan and others claim that it does; and if electricity does, why not employ it? I think it clearly evident that it performs its work in a very short space of time, thereby obviating the suspense incident to the anxious watching and waiting for the *uncertain* results of remedies given internally, and various other methods resorted to in such cases.

We understand and appreciate the value of the tampon, the use of ergot of rye, abdominal manipulation with cold, the hot douche, etc., but they all sometimes fail, and we are sorely perplexed to know the next best thing to do. I think the battery excells everything else in such troublesome cases, and that every physician should have one and understand how to use it. I don't wish to be understood that this is my hobby, but I do say that it is all I want in obstetric practice, and when once used on the parturient female, she, as a rule will call for it at her subsequent confinements. I am engaged for two particular cases now, and they have both sent me word not to fail to bring my battery when I am wanted.

In conclusion, I will ask, would my patient have survived if I had not had the battery? It is my honest opinion that she would not. The heart's action had grown so feeble that her extremities were perfectly cold, syncope quite marked. In the first place she lost entirely too much blood before I had an opportunity to use electricity, and in the second place she could never have survived the second violent hemorrhage, because of the depressed condition of the system from the first prolonged attack, if it had not been so promptly arrested by it. This afforded an opportunity for the use of restoratives, and in thus reviving her, true labor pains set in which kept up regularly from the stimulus given the genito-spinal nerves by the current, terminating the case so favorably without the use of instruments.

I am indebted to Dr. W. T. Baird of Mexico, (formerly associated with me in the practice of medicine at this place) for what little I know about electricity in the lying-in-chamber. As to its utility in other branches, I have but little to say, as I have not devoted the time and study to it, that he has.

I could mention a good many cases of post partem hemorrhage that yielded to the electric current, in, from one to five minutes, and all I have to say to my medical friends, is, to try it and they will not be disappointed.

